# Compound-specific stable isotope analyses in Falkland Islands seabirds reveal seasonal changes in trophic positions

**DOI:** 10.1186/s12898-020-00288-5

**Published:** 2020-04-15

**Authors:** Petra Quillfeldt, Juan F. Masello

**Affiliations:** Department of Animal Ecology & Systematics, Justus Liebig University Giessen, Heinrich-Buff-Ring 26, 35392 Giessen, Germany

**Keywords:** Compound-specific stable isotope analyses of amino acids, Gentoo penguin *Pygoscelis papua*, Magellanic penguin *Spheniscus magellanicus*, Southern rockhopper penguin *Eudyptes chrysocome*, Thin-billed prion *Pachyptila belcheri*, Wilson’s storm-petrel *Oceanites oceanicus*, Falkland/Malvinas archipelago

## Abstract

**Background:**

While nitrogen and carbon stable isotope values can reflect ecological segregation, prey choice and spatial distribution in seabirds, the interpretation of bulk stable isotope values is frequently hampered by a lack of isotopic baseline data. In this study, we used compound-specific isotope analyses of amino acids (CSIA-AA) to overcome this constraint and to study interspecific differences, seasonal and historical changes in trophic positions of five seabird species, three penguins and two petrels, from a sub-Antarctic seabird community.

**Results:**

CSIA-AA allowed comparing trophic positions of seabirds with temperate and polar distributions. Gentoo and Magellanic penguins had the highest trophic positions during the breeding season (3.7 and 3.9), but decreased these (2.9 and 3.3) during the feed-up for moult. Intra-specific differences were also detected in Thin-billed prions, where carbon isotope values clearly separated individuals with polar and temperate distributions, both in the breeding and interbreeding periods. Thin-billed prions that foraged in polar waters had lower trophic positions (3.2) than conspecifics foraging in temperate waters (3.8). We further investigated historical changes by comparing museum samples with samples collected recently. Our pilot study suggests that Rockhopper penguins, Magellanic penguins and Thin-billed prions with temperate non-breeding distributions had retained their trophic levels over a 90–100 year period, while Gentoo penguins and Thin-billed prions with polar non-breeding distributions had decreased trophic levels compared to historical samples. In contrast, Wilson’s storm-petrels had slightly increased trophic levels compared to samples taken in 1924–1930.

**Conclusions:**

We applied compound-specific stable isotope analyses across a range of contexts, from intra-specific comparisons between stages of the breeding cycle to inter-specific seabird community analysis that would not have been possible using bulk stable isotope analyses alone due to differences in isotopic baselines.

## Background

Nitrogen and carbon stable isotope values have widely been applied to study trophic positions and spatial distribution of animals, respectively [1]. Due to different properties of the tissues (e.g. inert vs. metabolically active tissues, different turn-over times), stable isotope analysis can potentially yield information on the foraging ecology of animals at different times of the annual cycle such as the moulting period [2, 3] or even enable us to look back into historical times through the analysis of museum samples [2, 4].

However, in certain contexts the interpretation of stable isotope values is hampered by a lack of isotopic baseline data. In particular, δ^15^N values from bulk stable isotope analysis cannot be compared directly among tissues derived from food webs in different areas. Trophic positions of predators can only be calculated from bulk nitrogen isotope values if bulk tissue δ^15^N values of lower trophic positions is known [4, 5]. However, there may be considerable temporal and spatial variability in the bulk tissue δ^15^N values of the primary producer or potential prey [6, 7]. Thus, the reference bulk tissue δ^15^N value of lower trophic positions has to match the predator tissue in space and time. For example, trophic positions can be estimated from bulk nitrogen values of feathers if reference bulk tissue δ^15^N values are available from the moult area and moulting season. In the case of long-distance migrants that moult their feathers in distant areas with difficult access during the moulting time, or in unknown places, this may not be possible. Consequently, it is usually incorrect to compare the trophic position of tissues grown at different sites (e.g. between two species with different migratory strategies) or at different times of the year (e.g. breeding and non-breeding season), unless proper baseline data are also collected.

For example, in an analysis of a seabird community at New Island, Falkland/Malvinas Islands, trophic positions of seabirds were compared based on feather samples [4]. However, this was only possible for birds with a relatively local distribution such as gulls, penguins and cormorants, while a pelagic seabird, the Thin-billed prion *Pachyptila belcheri*, had to be excluded because baseline data (e.g. krill stable isotope values) from their foraging grounds were not available. Likewise, very few samples that could be used as isotopic baseline for historical samples are kept at public (e.g. museums) or private institutions.

However, compound-specific stable isotope analyses of amino acids (CSIA-AA) [8] can be used in such cases to determine trophic positions directly from the tissues of the animals. The CSIA-AA method referred to here is the analysis of nitrogen isotope values in amino acids for the determination of trophic positions (reviewed in [9]). The method is based on the principle that certain amino acids, such as phenylalanine, do not fractionate with trophic position. These “source amino acids” (e.g. phenylalanine) therefore directly represent the primary producer at the base of the food web, and effectively provide the isotopic baseline needed to calculate the trophic position [9]. In contrast, other amino acids change largely between food and consumer, and are therefore called “trophic amino acids” (e.g. glutamic acid). Thus, the trophic position can be obtained from the difference in the nitrogen stable isotope values of glutamic acid and phenylalanine [8].

Previous studies have demonstrated that CSIA-AA data can provide a good estimate of the trophic position of marine organisms even from temporally and spatially variable environments. For example, the trophic position estimated by CSIA-AA in zooplankton near Hawaii was consistent over a period of 10 years, despite a temporal variance in bulk tissue δ^15^N values by up to 10‰ [6]. CSIA-AA has also been applied to determine the trophic positions of crustaceans [10, 11], fish [12] and seabirds [13, 14]. We previously applied this method to study the trophic segregation of sympatrically breeding storm-petrel species *Fregetta tropica* and *Oceanites oceanicus* that moult in pelagic waters [15].

In the present study, we aimed to study interspecific differences, seasonal and historical changes in the bulk stable isotope values as well as trophic positions determined by CSIA-AA of five seabird species (3 penguins and 2 petrels) breeding at the Falkland/Malvinas Islands and showing different distributions during the breeding and non-breeding period.

These species have contrasting foraging and distribution patterns, ranging from large diving, coastal penguins reaching benthic, high trophic-level prey to small, surface-feeding petrels that mainly feed on macrozooplankton (Table 1). Moreover, the penguins moult on land, while the petrels move to offshore waters for moult. Therefore, we aimed to test the following hypotheses:

1. Trophic positions determined by CSIA-AA reflect the known dietary differences among the species.
2. Because species segregate ecologically and are adapted to forage efficiently on specific prey, trophic positions are maintained within species throughout the year, in the breeding as well as the moulting season.
3. Because a regime shift has not been reported from the marine ecosystem around the Falkland Islands, trophic positions have not changed historically.

**Table 1:** Parameters of distribution and foraging of the five study species

Species	Diet	Distribution (breeding season)	Dive capacity	Distribution (moulting season)	Time of moult
Gentoo penguin	Variable, e.g. 40% fish, 60% lobster krill^b^, or over 80% fish^c^	Nearshore (30 km)^d^	120 m^d^	Unknown (suspected Falkland waters)	February–March^a^
Magellanic penguin	40% fish, 30% squid, 30% lobster krill^b^	Nearshore (40 km)^d^	70 m^d^	Unknown (suspected Falkland waters)	February–March^a^
Rockhopper penguin	50% krill, 50% small squid^b^	Nearshore (30 km)^d^	50 m^d^	Unknown (suspected Falkland/Patagonian Shelf waters)	February–March^a^
Thin-billed prion	Amphipods, small squid^b,e^	Offshore (500 km)^f^	< 10 m^g^	90% of population offshore Atlantic, around polar front, 10% over Patagonian shelf ^h^	April–May
Wilson’s storm-petrel	Mainly krill, variable amounts of small fish and squid^g^	Offshore (250 km)^i^	Surface feeding^g^	Temperate to sub-tropical^g^	April–May

^a^Own observation, References: ^b^Thompson [20], ^c^Handley et al. [22], ^d^Masello et al. [19], ^e^Quillfeldt et al. [18], ^f^Quillfeldt et al. [46], ^g^Brooke [47], ^h^Quillfeldt et al. [24], ^i^Croxall & Prince [48]

## Results

### Interspecific differences—breeding season

**Fig. 1:**
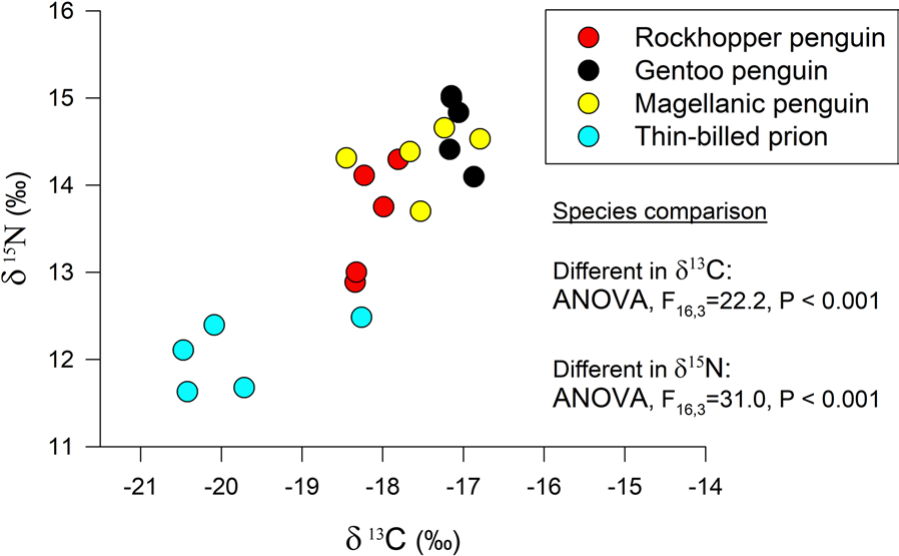
Bulk stable isotope values of carbon and nitrogen of four seabird species breeding at New Island, during the breeding season (blood samples)

Bulk stable isotope values in blood samples collected during the breeding season differed among the four sympatric seabird species (Fig. 1, Table 2). Thin-billed prions had clearly lower carbon stable isotope values. Smaller differences were observed between Rockhopper and Gentoo penguins, while Magellanic penguins had an intermediate carbon stable isotope value. Carbon and nitrogen bulk tissue stable isotope values correlated positively (Fig. 1).

**Table 2:** Bulk and compound-specific stable isotopic analyses of inter-specific differences of seabirds during the breeding season (based on red blood cell samples)

Species	N	δ^13^C (‰)	δ^15^N (‰)	Trophic position	δ^15^N [Glx] (‰)	δ^15^N [Phe] (‰)
Gentoo penguin	5	− 17.1 ± 0.4^b^	14.7 ± 0.1^b^	3.7 ± 0.2^a,b^	27.7 ± 1.5^b^	7.5 ± 0.2^b^
Magellanic penguin	5	− 17.5 ± 0.4^a,b^	14.3 ± 0.6^a,b^	3.9 ± 0.1^a^	26.2 ± 0.3^b^	6.9 ± 0.8^b^
Rockhopper penguin	5	− 18.1 ± 0.6^a^	13.6 ± 0.2^a^	3.4 ± 0.1^c^	23.3 ± 0.6^a^	7.2 ± 0.9^b^
Thin-billed prion	5	− 19.8 ± 0.4^c^	12.1 ± 0.9^c^	3.5 ± 0.1^b,c^	22.1 ± 1.1^a^	5.4 ± 0.4^a^
ANOVA		*F*_16,3_ = 22.2, *P* < 0.001	*F*_16,3_ = 31.0, *P* < 0.001	*F*_16,3_ = 12.9, *P* < 0.001	*F*_16,3_ = 19.4, *P* < 0.001	*F*_16,3_ = 12.0, *P* < 0.001

Bulk stable isotope values δ^13^C and δ^15^N, trophic positions calculated from CSIA-AA and δ^15^N values of the trophic amino acid Glx (glutamic acid) and the source amino acid Phe (phenylalanine). Means and standard deviations are given, and homogenous subgroups according to post hoc tests (TUKEY) are marked by similar letters

In compound-specific analyses, Thin-billed prions were also distinguished from the penguins by lower δ^15^N values of the source amino acid (phenylalanine, Table 2). In the trophic amino acid (glutamic acid) two groups were statistically distinguished, the Thin-billed prions and Rockhopper penguins had lower values than the Magellanic and Gentoo penguins (Table 2). Trophic positions ranged at 3.4 and 3.5 for the plankton feeding seabirds and 3.7 and 3.9 for the piscivores (Table 2), and the difference was statistically significant.

### Interspecific differences—inter-breeding season

Bulk stable isotope values in feather samples reflected spatial and dietary segregation during the pre-moult (penguins) and moulting (flying birds) season among the five seabird species (Table 3). Thin-billed prions also had clearly lower carbon stable isotope values during moult than the other species. In compound-specific analyses, Thin-billed prions were also distinguished from the other species by very low δ^15^N values (range − 1.6 to 5.3‰) of the source amino acid (phenylalanine, Table 3). Wilson’s storm-petrels and Rockhopper penguins had intermediate values, while Magellanic and Gentoo Penguins had the highest stable isotope values (e.g. range in Gentoo Penguins 8.6 to 11.3‰) in the source amino acid. In the trophic amino acid (glutamic acid), large variances were observed. The trophic positions were lowest in the penguins, intermediate in Thin-billed prions and highest in the Wilson’s storm-petrels (Table 3).

**Table 3:** Bulk and compound-specific stable isotopic analyses of inter-specific differences of seabirds during moult (based on feather samples)

Species	N	δ^13^C (‰)	δ^15^N (‰)	Trophic position	δ^15^N [Glx] (‰)	δ^15^N [Phe] (‰)
Gentoo penguin	5	− 17.1 ± 0.4^a^	14.7 ± 0.1^a^	2.9 ± 0.2^a^	22.8 ± 1.0^a,b^	10.1 ± 1.0^a^
Magellanic penguin	5	− 17.5 ± 0.4^a^	14.3 ± 0.6^a^	3.3 ± 0.3^a,b^	23.7 ± 1.8^a^	9.0 ± 0.8^a^
Rockhopper penguin	5	− 18.1 ± 0.6^a^	13.6 ± 0.2^a,b^	3.2 ± 0.7^a,b^	22.5 ± 1.5^a,b^	8.0 ± 1.5^a,b^
Thin-billed prion (all)	11	− 20.8 ± 3.1^b^	11.9 ± 4.1^b^	3.5 ± 0.4^b,c^	18.6 ± 4.6^b^	2.2 ± 2.7^c^
Wilson’s storm-petrel	4	− 17.4 ± 0.6^a^	16.1 ± 1.2^a^	4.0 ± 0.1^c^	24.4 ± 1.1^a^	5.3 ± 1.0^b^
ANOVA		*F*_25,4_ = 7.2, *P* < 0.001	*F*_25,4_ = 4.1, *P* = 0.011	*F*_25,4_ = 8.8, *P* < 0.001	*F*_25,4_ = 4.3, *P* < 0.001	*F*_25,4_ = 21.4, *P* < 0.001

Bulk stable isotope values δ^13^C and δ^15^N, trophic positions calculated from CSIA-AA and δ^15^N values of the trophic amino acid Glx (glutamic acid) and the source amino acid Phe (phenylanaline). Means and standard deviations are given, and homogenous subgroups according to post hoc tests (TUKEY) are marked by similar letters

### Seasonal changes in penguins

**Fig. 2:**
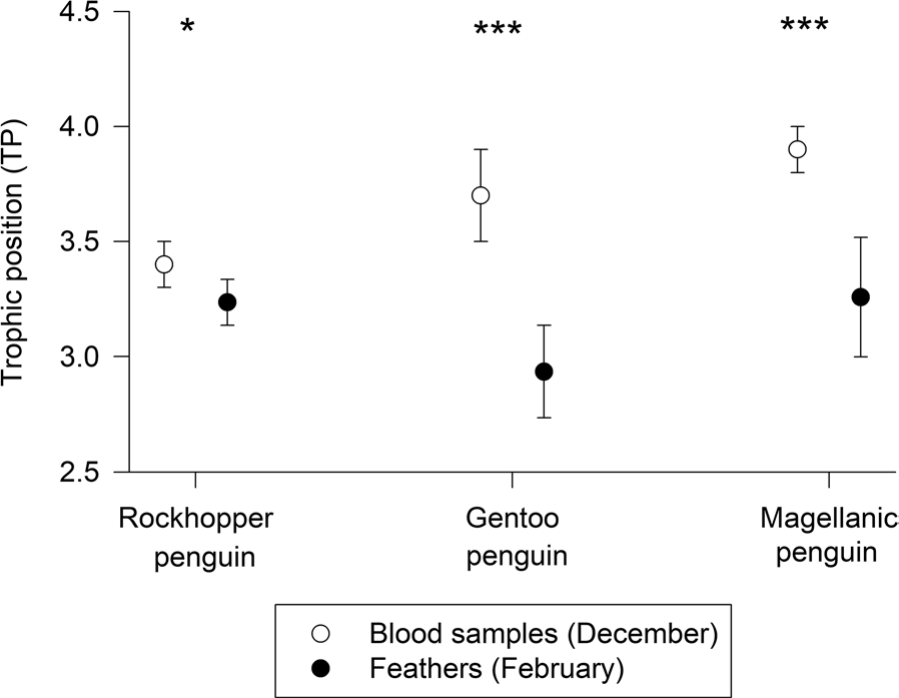
Trophic positions calculated from CSIA-AA of three penguin species breeding at New Island, during the breeding season (blood samples) and during moult (feather samples). Blood and feather values were compared using t-tests, and significance levels are marked with asterisks (* for P < 0.05, and *** for P < 0.001)

A comparison between feather and red blood cell stable isotope values indicated lower trophic positions during moult than during the chick-rearing season, in all three penguin species (Fig. 2). The differences were highly statistically significant in Magellanic penguins (*t* test, t = − 5.2, d.f. = 4.8, *P* = 0.002) and Gentoo Penguins (*t* = − 6.1, d.f. = 7.8, *P* < 0.001), and statistically significant in Rockhopper penguins (*t* = − 2.4, d.f. = 8.0, *P* = 0.021). These differences were caused by lower δ^15^N values of the source amino acid (phenylalanine) and higher values in the trophic amino acid (glutamic acid) during the chick-rearing season (Tables 2 and 3). The differences in δ^15^N values of phenylalanine were statistically significant for Gentoo Penguins (*t* = − 5.5, d.f. = 4.4, *P* = 0.004) and Magellanic penguins (*t* = − 4.1, d.f. = 8, *P* = 0.003), but not for Rockhopper penguins (*t* = − 1.0, d.f. = 6.4, *P* = 0.333). Likewise, the differences in δ^15^N values of glutamic acid were statistically significant for Gentoo penguins (*t* = 3.7, d.f. = 7, *P* = 0.008) and Magellanic penguins (*t* = 3.0, d.f. = 4.3, *P* = 0.038), but not for Rockhopper penguins (*t* = 1.1, d.f. = 5.5, *P* = 0.311). Gentoo penguins had a notably low trophic position (2.9) during moult according their feather values (Fig. 2).

### Seasonal changes in Thin-billed prions

During the breeding season, we obtained trophic positions ranging from 3.2 in egg membranes to 3.9 in adult Thin-billed prions foraging predominantly in temperate waters (Table 4). The differences in trophic positions were statistically significant (ANOVA, *F*_19,4_ = 6.1, *P* = 0.002), with higher TP values in adults (represented by induced feathers) and lower TP in investments into offspring (egg membranes, chick feathers and chick blood cells, Table 4).

**Table 4:** Intra-specific differences in bulk and compound-specific stable isotopic analyses of Thin-billed prions *Pachyptila belcheri*

Tissue	*n*	δ^13^C (‰)	δ^15^N (‰)	Trophic position	δ^15^N [Glx] (‰)	δ^15^N [Phe] (‰)
***Breeding season***						
Induced feathers						
All	8	− 18.5 ± 2.6	13.0 ± 1.6	3.9 ± 0.2	21.2 ± 2.4	3.3 ± 2.1
Polar	2	− 22.5 ± 0.2	10.7 ± 1.1	3.7 ± 0.1	18.4 ± 0.6	0.9 ± 0.4
Temperate	6	− 17.2 ± 0.9	13.7 ± 0.6	3.9 ± 0.2	22.7 ± 1.5	4.1 ± 1.8
Chick blood cells	5	− 19.8 ± 0.9	12.1 ± 0.4	3.5 ± 0.1	22.1 ± 1.1	5.4 ± 0.4
Chick feathers	5	− 18.5 ± 1.0	13.7 ± 0.4	3.4 ± 0.1	21.3 ± 0.7	5.6 ± 0.4
Egg membranes	6	− 16.4 ± 0.7	11.7 ± 0.9	3.2 ± 0.4	19.8 ± 3.3	5.5 ± 0.6
***Inter-breeding season (moult, based on tail feathers)***						
Polar moulting	5	− 24.0 ± 0.4	7.8 ± 0.4	3.2 ± 0.1	14.3 ± 1.5	0.0 ± 1.8
Temperate moulting	6	− 18.2 ± 0.9	15.4 ± 1.3	3.8 ± 0.3	22.1 ± 2.8	4.0 ± 1.8

Bulk stable isotope values δ^13^C and δ^15^N, trophic positions calculated from CSIA-AA and δ^15^N values of the trophic amino acid Glx (glutamic acid) and the source amino acid Phe (phenylanaline). Means and standard deviations are given

**Fig. 3:**
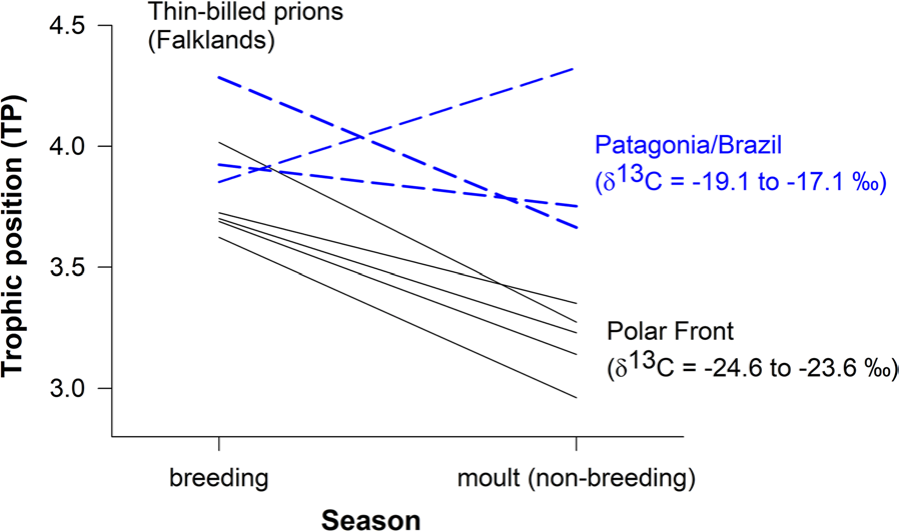
Trophic positions calculated from CSIA-AA of Thin-billed prion feather samples grown during the breeding season (induced feathers) and the non-breeding season (moult). Bulk carbon stable isotope values were used to distinguish polar and temperate moulting grounds (polar for δ^13^C < − 21‰). Black lines represent individuals migrating to Polar waters while blue lines represent individuals migrating to the Patagonian Shelf or areas influenced by the Brazilian Current

Furthermore, Thin-billed prions that foraged in polar waters had lower trophic positions than conspecifics foraging in temperate waters with polar distribution in the breeding season (3.7 vs. 3.9, t = − 4.6, df = 5.8, *P* = 0.002) as well as during moult (3.2 vs. 3.8, t = − 4.6, df = 5.8, *P* = 0.002, Table 4). The paired analyses of induced and original feathers showed that seven of eight individuals decreased the trophic position during the moulting period compared to the breeding season (Fig. 3, paired t-test: *t* = 2.8, d.f. = 7, *P* = 0.013).

### Historical changes in trophic positions

**Fig. 4:**
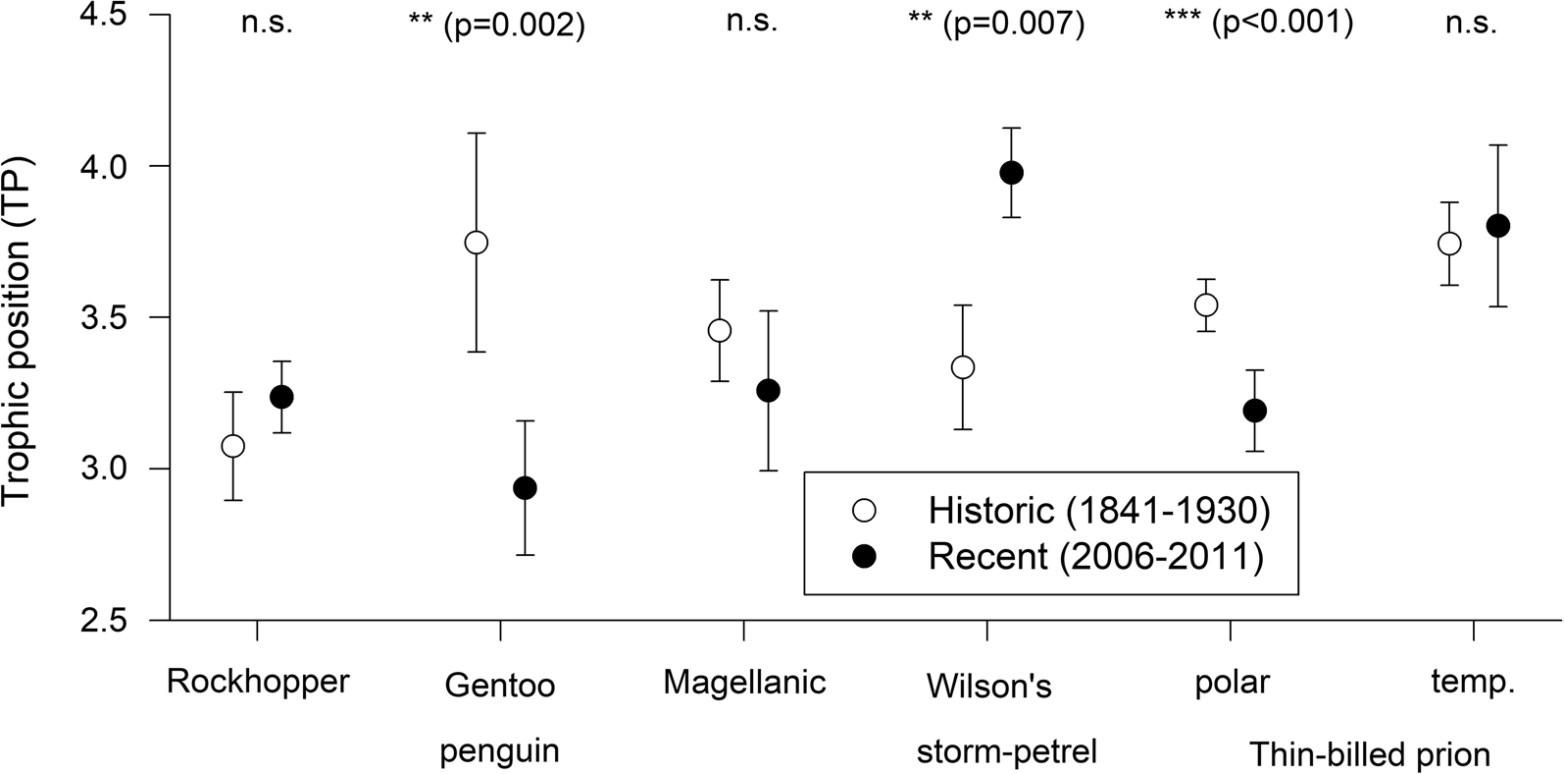
Trophic positions calculated from CSIA-AA of seabird feather samples grown during the non-breeding season (moult) in recent and historical samples. Bulk carbon stable isotope values were used to distinguish polar and temperate moulting grounds (polar for δ^13^C < − 21‰) in Thin-billed prions. Recent and historical samples were compared using t-tests, and significance levels are marked with asterisks (** for P < 0.01, and *** for P < 0.001)

**Fig. 5:**
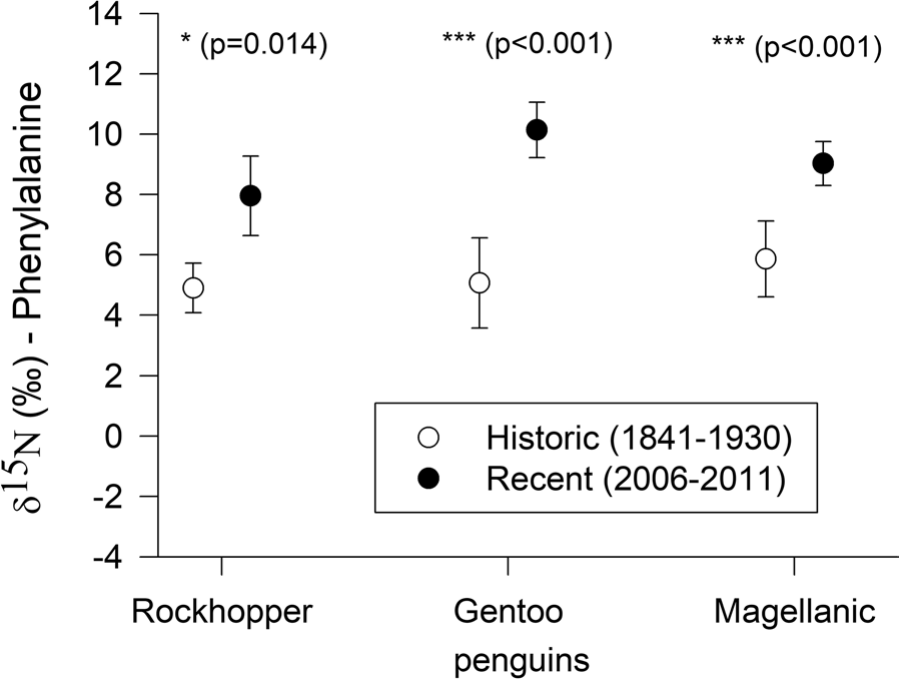
Historical changes δ^15^N in the source amino acid (phenylalanine) of feathers grown during the non-breeding season (moult) in recent and historical samples of three penguin species breeding at New Island, Falkland/Malvinas Islands. Recent and historical samples were compared using t-tests, and significance levels are marked with asterisks (* for P < 0.05, and *** for P < 0.001)

Among six groups of seabirds, three (Thin-billed Prions moulting in temperate waters, Rockhopper and Magellanic penguins) retained their trophic positions over time (Fig. 4, Table 5). However, differences were observed in three species: Gentoo penguins formerly (1915) had higher trophic positions in adult feathers (3.75 ± 0.36) than recently (2.93 ± 0.22). These differences were caused by an increase in phenylanaline δ^15^N values (10.1 ± 0.9‰ vs. 5.1 ± 1.5‰) in recent feathers compared to historical feathers (Fig. 5), indicating a change in the base of the food web or a change to a different foraging habitat. An increase in phenylanaline δ^15^N values was also observed in the other two penguin species (Table 5, Fig. 5).

**Table 5:** Historical changes, based on recent and museum feather samples, in bulk stable isotope values, in trophic positions calculated from CSIA-AA and δ^15^N values of the trophic amino acid Glx (glutamic acid) and the source amino acid Phe (phenylanaline)

Species	δ^13^C	δ^15^N	Trophic position	δ^15^N [Glx]	δ^15^N [Phe]
Rockhopper penguin	ns	*t* (5.6) = 5.3, *P* = 0.002	ns	*t* (5.9) = 5.3, *P* = 0.002	*t* (5.7) = 3.5, *P* = 0.014
Gentoo penguin	*t* (8.0) = − 3.6, *P* = 0.007	*t* (7.7) = 3.0, *P* = 0.018	*t* (6.6) = − 4.3, *P* = 0.002	ns	*t* (6.6) = 5.8, *P* < 0.001
Magellanic penguin	ns	ns	ns	ns	*t* (6.4) = 4.4, *P* = 0.004
Thin-billed prion					
Polar	*t* (8.0) = − 2.5, *P* = 0.035	*t* (4.4) = − 7.3, *P* = 0.001	*t* (6.9) = − 5.0, *P* < 0.001	*t* (5.4) = − 2.9, *P* = 0.032	ns
Temperate	ns	ns	ns	ns	ns
Wilson’s storm-petrel	ns	ns	*t* (4.6) = 3.5, *P* = 0.007	ns	ns

The number behind the test statistic *t* in parenthesis is the degrees of freedom (d.f.)

In Thin-billed prions, those individuals that moulted in polar waters decreased their trophic positions (from 3.54 ± 0.09 to 3.19 ± 0.13) but here, the change was observed in the trophic amino acid (glutamic acid, Table 5).

Finally, Wilson’s storm-petrels sampled in 1924–1930 had lower trophic levels (3.33 ± 0.20‰) than those sampled recently (3.98 ± 0.15‰). In this species, no significant changes were observed in either the phenylanaline or glutamic acid δ^15^N values. However, while glutamic acid δ^15^N values were clearly very similar (t-test, *t* = 0.1, d.f. = 2.2, *P* = 0.937), phenylanaline δ^15^N values tended to be lower in recent samples (9.5 ± 2.7‰ vs. 5.3 ± 0.8‰, *t* = 2.1, d.f. = 2.2, *P* = 0.077).

## Discussion

We applied CSIA-AA to study interspecific differences, seasonal and historical changes in five seabird species (three penguins and two petrels) breeding at the Falkland/Malvinas Islands. Although previous studies have applied stable isotope analyses to study this seabird community [4], this was done separately for diving species (e.g. [4, 16] and the pelagic petrels [17] due to the very different distribution. For the same reason, these analyses focussed to the breeding season. In these contexts, the CSIA-AA method facilitates the estimation of trophic positions independently of baseline samples.

Across the study, we observed large differences in δ^15^N values of the source amino acid phenylalanine (range − 1.6 to 11.9‰). Thin-billed prions had very low phenylanaline δ^15^N values, and this was especially pronounced in individuals with a polar distribution according to bulk carbon isotope values, with values in feathers around zero (range − 1.6 to 2.1‰). Thin-billed prions feed mainly offshore in oceanic waters on zooplankton, especially krill and amphipods [18] which they take at or close to the sea surface. In contrast, Gentoo penguins that feed nearshore and take a high percentage of benthic prey (e.g. [19], Table 5), had high phenylanaline δ^15^N values around 10‰ in their feathers (range 8.6 to 11.3‰). These large differences in nitrogen stable isotope values of phenylalanine as source amino acid reflect the isotopic differences between the offshore-pelagic and the nearshore-benthic food web and were in line with differences observed in bulk tissue δ^13^C values between the species.

### Interspecific and seasonal differences

Our calculated trophic positions for different species during the breeding season, based on differences in the δ^15^N values of amino acids of red blood cells, were in line with results from conventional diet analyses (e.g. [20]). Planktonic predators had lower mean values than fish predators (Table 2): Thin-billed prions and Rockhopper penguins feed largely on crustaceans and small squid [18, 20, 21] and had trophic positions around 3.2–3.3 (Table 2), while Magellanic and Gentoo penguins that take more fish [20–22] had trophic positions around 3.7–3.9.

However, surprisingly during moult we found a different pattern, as the trophic positions were lowest in the penguins, intermediate in Thin-billed prions and highest in Wilson’s storm-petrels (Table 3). This pattern was caused by a decrease in the trophic position of the two piscivorous penguin species. Penguins carry out a complete moult shortly after the breeding season. After the chicks fledge, adults accumulate fat during a short period of intense foraging, and then return to the colonies to moult during a period on land when they are not able to forage. Thus, the diet during the feed-up for moult will depend on the diet available during this short time window. Wilson’s storm-petrels migrate to northern latitudes, where food chains may be more complex and have also been reported to scavenge at natural oil slicks and carrion, and to follow ships to pick at offal (https://www.audubon.org/field-guide/bird/wilsons-storm-petrel).

Especially low TP values were observed in feather of Gentoo penguins that are opportunistic foragers, using variable amounts of benthic and pelagic foraging according to food availability [19, 23]. In poor seasons, Gentoo penguins can take considerable amounts of Lobster Krill *Munida gregaria* instead of fish [20]. The feathers analysed here were collected in a single season and especially in the opportunistic species like Gentoo penguins, inter-annual comparisons of trophic positions calculated with CSIA-AA would be interesting. Both the composition and the size of fish may change within and among seasons in Gentoo penguin diets [11, 21], and this can influence the trophic position. Previous conventional studies of Gentoo penguin diets in the Falkland/Malvinas Islands have shown a large range of possible trophic positions, from birds feeding mainly on *Munida gregaria* (low trophic position, e.g. δ^15^N_Bulk_ values of 10.0‰ and 9.3‰ during two surveys [7]), and Patagonian rock cod *Patagonotothen ramsayi* (high trophic position, e.g. δ^15^N_Bulk_ values of 13.5‰, 12.8‰ at the same time [7]). Therefore, the relatively large changes in trophic positions in this species are plausible.

Seasonal changes in trophic positions were also observed in Thin-billed prions. Like the penguins, prions also had lower trophic positions during moult. However, in the prions, moult is associated with a movement away from the colony, to oceanic waters. Over 90% of the population migrates in eastward direction and moults in oceanic waters around the polar front [24]. These Thin-billed prions had lower trophic positions during moult (2.9) than conspecifics foraging in temperate waters (4.0, Table 4), and all polar moulting individuals decreased the trophic position during the moulting period compared to the breeding season (Fig. 3). The diet during the breeding season (TP 3.9) consists of crustaceans and small squid, with a special preference for the amphipod *Themisto gaudichaudii* [17]. The trophic position of this amphipod has been determined using the CSIA-AA method near the Antarctic Polar Front [25], where the two forms of *T. gaudichaudii* were found to feed at different trophic positions, with *T. gaudichaudii bispinosa* feeding at a higher trophic position (TP 3.3) than *T. gaudichaudii compressa* (TP 2.8). The trophic position values (mean TP 3.9) observed in the present study thus correspond well to the observed amphipod-based diet of Thin-billed prions during the breeding season [18].

We further observed a difference in trophic position between adult Thin-billed prions and eggs and chicks (Table 4). Such differences in seabird diets for self-feeding and chick provisioning have been demonstrated both with conventional methods and stable isotope analyses [26–28], and are probably related to a trade-off between the mass of food carried to feed chicks and the distance travelled to catch prey [26].

### Historical changes in trophic positions

Due to very small sample sizes, the observed changes in stable isotope values will require further support should more historical material become available. In the penguins, an increase in phenylanaline δ^15^N values was observed in all three species (Fig. 5), associated with a decrease in calculated trophic positions in Gentoo penguins. Most historical samples come from the East Falkland area (Additional file 1: Table S1). In present studies, East Falkland Gentoo penguins tend to have higher benthic fish contents in their prey, especially of Rock cod [22] than West Falkland samples [20], which are more frequently dominated by the pelagic Falkland herring and lobster krill. According to the present data, pelagic species show lower δ^15^N values of phenylalanine and thus, lower values would be expected in the West Falkland (recent New Island) Gentoo penguin samples. However, we here observed the opposite trend, and therefore we think that locality alone cannot explain the differences. In any case, a comparison with recent samples from the East Falkland Islands would be interesting.

The difference in phenylanaline δ^15^N values suggests isotopic differences at the base of the food web (i.e. the isotopic baseline), that can be caused by a change in the primary producers or a difference in the habitat use (offshore vs. nearshore and/or benthic vs. pelagic). Because all three species were affected in a similar manner, a change in the food web is more likely than a behavioural change. Fast or slow changes across ecosystems, known as regime shifts, have been observed in response to eutrophication and overfishing or removal of apex predators in several nearshore marine ecosystems, such as the transformation of coral-dominated reefs to algae-dominated reefs or the establishment of sea-urchin dominance in kelp forests [29]. Due to climate change, Australian temperate reef communities lost their defining kelp forests and became dominated by persistent seaweed turfs [30]. Although such changes in Falkland/Malvinas Islands marine habitats have not been reported, the present data suggest that some change at the base of the food web has occurred over the last 90–100 years. The Falkland/Malvinas Islands are situated in the waters of the Patagonian Shelf, one of the widest shelves in the world, supporting a highly productive marine ecosystem that supports important seabird colonies (e.g. [31]). In the southern part of the Patagonian Shelf, south of and around the Falkland/Malvinas Islands, high primary productivity is supported by upwelling of cold Antarctic waters. Year-round and seasonal tidal fronts, such as the Bahía Grande Front and the Valdés Front play an important role in ecological processes, allowing for high biological production, offering feeding and/or reproductive habitats for fishes, squids, and birds [31]. Changes in the current systems associated with warmer sea surface temperatures have been found to influence the food availability both for pelagic and nearshore foraging seabirds [18, 32, 33]. Hilton et al. [34] compared bulk isotopic values in historical and recent feather samples of rockhopper penguins from seven breeding sites across the sub-Antarctic and found decreases in bulk tissue δ^13^C values which may indicate decreasing primary productivity, associated with the decline of penguin populations. There was some evidence of a long-term decline in bulk tissue δ^15^N values at some sites, and δ^15^N values were negatively related to sea surface temperatures, which may indicate of a shift to lower trophic position prey over time and in warm years in this species [34]. However, samples of the Falkland/Malvinas Islands had not been included in these analyses.

In Thin-billed prions, previous studies have reported a change in the frequency of adult migration patterns, with a predominance of adults migrating to polar waters for moult in recent years [2]. As mentioned above, this corresponds to 90% of the recent population migrating in eastward direction to moult in oceanic waters around the polar front [24]. The present study suggests that those individuals that moulted in polar waters decreased their trophic positions (from 3.6 to 2.9). This change was caused by a decrease in the stable isotope values of the trophic amino acid (glutamic acid, Table 5), thus indicating no major differences at the base of the food web but rather a difference in the food taken. The food available at such a low trophic position (1.9) could likely consist of very small zooplankton such as copepods or larval krill that have been found in the diet of Thin-billed prions during breeding season with poor food availability [18].

Finally, Wilson’s storm-petrels sampled in 1924–1930 had lower trophic levels (3.2) than those sampled recently (4.3). In this species, phenylanaline δ^15^N values tended to be lower in recent samples (9.5 vs. 5.3), a trend contrasting with the increase observed in the penguins. However, Wilson’s storm-petrels are highly mobile species that can migrate from the Antarctic to the northern hemisphere, and this change may therefore correspond to changes in the winter distribution, as observed in other Southern Ocean petrels [2].

This study has a number of limitations, namely low sample sizes per group and the use of the same trophic discrimination factor for all species. The low sample sizes are mainly attributable to the high costs and time involved in the analyses and this also restricted the number of years and study sites included. Studies of multiple years and sites would now be needed in order to consolidate the patterns observed here.

## Conclusions

In summary, we applied CSIA-AA data across a range of contexts, from intra-specific comparisons between stages of the breeding cycle to inter-specific seabird community analysis that would not have been possible using bulk stable isotope analyses alone due to differences in isotopic baselines.

Historical changes suggested that some change at the base of the food web has occurred over the last 90–100 years, which may need further investigation. These findings will hopefully stimulate the search for other material, e.g. plankton samples, to study the changes in the food web. Furthermore, the full potential of the CSIA-AA method may be seen in combination with other methods generating positional (GPS or geolocation) or dietary data, which will allow us to gain a more complete understanding of the differences in trophic positions. In addition, the distribution of source amino acid stable isotope values among primary producers in different food webs and environmental conditions merits further study, especially in extreme environments such as the open oceans and polar waters. Such data are needed to explain the underlying mechanisms of the patterns observed here. The determination of the trophic discrimination factor for more specific groups such as species and stages (e.g. adults versus chicks) would also further increase the accuracy of the method, but requires captive studies.

## Methods

### Sample collection

Samples were collected at New Island and Beauchêne Island, Falkland/Malvinas Islands (51°43′S, 61°17′W) during the breeding seasons 2006–2007 and 2008–2009 (New Island) and in November 2011 (Beauchêne Island). Both islands are recognized as important bird areas for their seabird colonies [16, 17, 35]. We included samples representing the moulting (flying birds) or pre-moulting (penguins) season (adult feathers) from all species, as well as samples from the breeding season (red blood cells) from Thin-billed prions and the three penguin species. Red blood cells represent the diet ingested ca. 2 to 4 weeks before the sampling [17]. Further samples from the breeding season (chick feathers, egg membranes and induced undertail covert feathers from adults) were analysed in Thin-billed prions. A total of 95 samples from five species of seabirds were analysed (Tables 1, 2, 3). At New Island, white feathers from penguins were collected non-invasively from birds during the moulting season, by checking the area around breeding colonies for moulted feathers. Feathers from penguins were collected during moult from the ground in front of individual burrows of Magellanic penguins *Spheniscus magellanicus* or around moulting groups of Southern Rockhopper penguins *Eudyptes chrysocome* and Gentoo penguins *Pygoscelis papua*. It is highly unlikely that two moulted feathers of penguins are from the same bird because several hundred to thousand birds moult in a group and we collected a single feather from each site, with a minimum distance of 20 m between collection sites. Egg membranes and chick blood samples from Thin-billed prions were collected during studies of the breeding biology of this species (e.g. [17]). Egg membranes represent the time of the pre-laying exodus, when the females gather nutrients to form the egg.

In Thin-billed prions, most birds (92%) moult in polar waters, while the remaining 8% moult in temperate waters [2]. From feathers analysed previously [17], we selected five birds with polar and six birds with temperate moulting grounds, using a cut-off value of the carbon isotope value of − 21‰ [3]. From eight of these birds (five with polar and three with temperate moult), we also obtained induced feathers by removing an undertail covert feather at the beginning of the breeding season in December and the regrown feather several weeks later in February. Regrowing feathers could be distinguished clearly, because regrowth was not yet complete at the time of sampling. Of these eight feathers, two were grown from resources collected predominantly in polar waters (bulk δ^13^C < − 21‰).

Blood samples of penguins were collected during a previous tracking study [16]. At Beauchêne, we collected outer tail feathers of Wilson’s storm-petrels caught by mistnet. All sampling was carried out under licence from the Falkland Islands Government Environmental Office.

We further investigated historical changes by comparing museum samples with samples collected recently. Samples were obtained from the American Museum of Natural History and the British Natural History Museum as described in [2]. Only samples that could at least be dated to a specific year where used in the analysis. All samples had been collected at the Falkland Islands (penguins and storm-petrels) or offshore in Patagonian shelf waters between Argentina and the Falkland Islands (prions). The samples (*n* = 10 for Thin-billed prions, *n* = 5 for Gentoo and Magellanic penguins and *n* = 3 for Rockhopper penguins and Wilson’s storm-petrels) were collected between 1915 and 1930, except for three older Rockhopper penguin samples (one from 1841 and two from 1876). Sample details are summarized in Additional file 1: Table S1.

### Stable isotope analyses

Bulk C and N stable isotope analyses and CSIA-AA analyses were conducted at the UC Davis Stable Isotope facility. Bulk carbon and nitrogen isotope analyses were carried out on 0.65–0.7 mg aliquots of feathers cut into fragments with stainless steel scissors, and weighed into tin cups. Lipids were not washed off the feathers as they were shown to have negligible effects on the isotope values [36]. Carbon and nitrogen isotope values were measured simultaneously by continuous-flow isotope ratio mass spectrometry (CF-IRMS), using a PDZ Europa ANCA-GSL elemental analyser interfaced to a PDZ Europa 20–20 isotope ratio mass spectrometer (Sercon Ltd., Cheshire, UK). Stable isotope values were expressed in δ notation as parts per mil (‰) relative to the international standards V-Pee Dee Belemnite for δ^13^C and to atmospheric N_2_ for δ^15^N values. Laboratory standard measurements, previously calibrated against NIST Standard Reference Materials, indicated standard deviations of < 0.3‰ for δ^13^C and δ^15^N values. Laboratory reference materials and expected values were Bovine Liver (δ^13^C = − 21.7‰, δ^15^N = 7.7‰), USGS-41 Glutamic Acid (δ^13^C = 37.6‰, δ^15^N = 47.6‰), Nylon5 (δ^13^C = − 27.7‰, δ^15^N = − 10.3‰), and Glutamic Acid (δ^13^C = − 16.7‰, δ^15^N = − 6.8‰). Specifically, SDs in the analytical run for the reference standards were 0.12 (δ^13^C) and 0.08 (δ^15^N) for Bovine Liver, 0.24 (δ^13^C) and 0.09 (δ^15^N) for USGS-41 Glutamic Acid, 0.15 (δ^13^C) and 0.13 (δ^15^N) for Nylon5 and 0.05 (δ^13^C) and 0.24 (δ^15^N) for Glutamic Acid.

Compound-specific ^15^N isotope analysis for amino acid derivatives followed Walsh et al. [37] and [38], using a Thermo GC-C-IRMS system composed of a Trace Ultra GC gas chromatograph (Thermo Electron Corp., Milan, Italy) coupled to a Delta V Plus isotope ratio mass spectrometer through GC IsoLink interface (Thermo Electron Corp., Bremen, Germany). 4 mg of feathers were cut into fragments with stainless steel scissors. Prior to chromatography, proteins were hydrolysed from feathers (6 M HCl, 70 min., 150 °C under N_2_ headspace) to enable their chromatographic separation in a DB-23 (Agilent Technologies) column (30 m, 0.25 mm O.D., 0.25 mm film; constant flow 1.6 mL/min). The derivatives are methoxycarbonyl amino acid methyl esters (MOC AA esters). Walsh et al. 2014 [37] demonstrated that, while there are two deriviatization products for glutamic acid (Glx) using chloroformates, if the pH is maintained below 1, the primary product is representative of the ^15^N of parent glutamic acid. MOC AA esters were thus derivatized in a buffer of 0.4 M HCl, such that for all natural materials, this maintains a pH ≪ 1. In a comparison of MOC AA esters versus a more conventional acetylation-esterification technique (*n*-acetyl isopropyl esters) across a range of sample materials [38], the differences between methods were less than expected measurement error, including for Glx. Once separated chromatographically, each compound was combusted at 1000 °C with Ni/NiO/CuO catalyst and introduced into the isotope ratio mass spectrometer. Compound identification was carried out by a Varian CP3800 gas chromatograph coupled to a Saturn 2200 ion trap MS/MS (Varian, Inc., Walnut Creek, CA U.S.A.). Internal and external standards were used as detailed previously [38]. Specific details for the analytical run carried out for this study are given in Additional file 1: Table S2. Measurements of reference material used for quality assurance had a mean deviation from the known value of 0.09‰ (maximum 0.51‰). As described previously [15], compound-specific nitrogen isotope analyses were carried out on duplicate samples and the repeatability was high with 96.6% for TP, 90.0% for glutamic acid and 89.8% for phenylalanine.

### Statistical data analysis

It has been suggested that trophic positions (TP) can be calculated from the nitrogen stable isotope values of glutamic acid (Glx) and phenylalanine (Phe), as TP = ((Glx-Phe-3.4‰)/7.6‰) + 1 [39]. The trophic discrimination factor (TDF_Glx-Phe_) of 7.6‰ is found in several marine trophic steps such as zooplankton consuming phytoplankton and fish consuming zooplankton [39] and appears robust for lower TP organisms (e.g. [40]). More recently, a meta-analysis has derived a lower TDF_Glx-Phe_ value of 6.2‰ [41]. Moreover, there is growing evidence that TDF_Glx-Phe_ values are not constant across all trophic positions (e.g. [42]). In birds lower TDF have recently been found in American kestrels *Falco sparverius* (4.1‰ for muscle and 5.4‰ for red blood cells) [43] and Gentoo penguins (3.5‰ for feathers) [44]. To analyse seabird feathers, it would therefore seem appropriate to use a multi-TDF_Glx-Phe_ [42]:

$${\text{TP}}\left[ {\text{feathers}} \right] = 2 + \frac{Glx - Phe - 3.5{\permil} - 3.4{\permil}}{6.2{\permil}}$$

where 6.2 is the overall mean TDF across a wide range of taxa, diet types, and modes of nitrogen excretion [41], 3.5 is the TDF for seabird feathers, and 3.4‰ is the difference in δ^15^N values between Glx and Phe in primary producers (plankton). For red blood cells we applied a slightly higher TDF of 4‰:

$$\text{TP}\left[ {\text{blood cells}} \right] = 2 + \frac{Glx - Phe - 4\permil - 3.4\permil}{6.2\permil}$$

We derived this value from a comparison of the TP values of feathers and red blood cells grown at the same time in Thin-billed prion chicks for different TDF, given that both tissues should reflect the same trophic position of the birds, and a similar time frame of 2–4 weeks [17]. Undertail covert feathers are 50–60 mm long and take about the same time to grow (own observation).

Data analyses were carried out in R 3.6.0. We tested for normality using Shapiro–Wilk tests and by checking plots of the data. We used the tsum.test function in the R package BSDA to perform one-sided t-tests and the aovSufficient function in the R package HH to perform ANOVAs on the means, standard deviations and sample sizes. To test for homogeneity of variance, we used Levene’s tests (function leveneTest in the R package car.Glx and Phe are the most commonly used amino acids to estimate trophic positions. However, some studies have used multiple trophic and source amino acids to estimate relative trophic level (e.g. [45], reviewed by [41]). Therefore, we calculated TP based on all available data, namely Phe as source amino acid, and the six trophic amino acids Glx, Ala, Leu, Ile, Pro and Asp. We obtained a very good correlation of the resulting TP values using either the glutamic acid and phenylalanine approach or the multiple approach (with quite similar means of 3.69 vs. 3.57, Additional file 1: Fig. S1).

We therefore decided to use the glutamic acid and phenylalanine approach, based on the fact that we have a poorer database on other AAs, compared to glutamic acid and phenylalanine. For example, trophic discrimination factors for other source and trophic amino acids specifically for e.g. blood and feathers are still lacking, and trophic enrichment is more variable (e.g. more differences among feeding types) in most other trophic amino acids, maybe with exception of proline [43].

## Supplementary information

**Additional file 1: Table S1.** Samples included in this study. All historical samples were collected in the Falkland/Malvinas area (^a^locality unknown, ^b^Stanley area and Beaver Island, East Falkland, ^c^offshore waters west of the archipelago, ^d^Grand Jason, West Falklands, ^e^Fox Bay, West Falklands) and are deposited in the American Museum of Natural History (AMNH) and the British Natural History Museum (BNHM). **Table S2.** Reference material values measured in the analytical run carried out for this study. **Fig. S1.** Comparison of trophic positions (TP) calculated usung the Glx and Phe approach versus the multiple amino acid approach.

## Data Availability

Raw data files will be available in the PANGAEA data archive upon publication, and are currently available from the corresponding author upon request.

## Abbreviations

CSIA-AA: Compound-specific isotope analyses of amino acids
CF-IRMS: Continuous-flow isotope ratio mass spectrometry
Glx: Glutamic acid
Phe: Phenylalanine
TDF: Trophic discrimination factor
TP: Trophic positions
