# Supplementary material for: Compound-specific stable isotope analyses in Falkland Islands seabirds reveal seasonal changes in trophic positions
Source: BMC Ecol. 2020 Apr 15;20:21. doi: 10.1186/s12898-020-00288-5 (PMC7160925; doi:10.1186/s12898-020-00288-5)

Additional file 1

Title: Compound-specific stable isotope analyses in Falkland Islands seabirds reveal seasonal changes in trophic positions

Authors: Petra Quillfeldt & Juan F. Masello

Table S1. Samples included in this study. All historical samples were collected in the Falkland/Malvinas area ([a] locality unknown, [b] Stanley area and Beaver Island, East Falkland, [c] offshore waters west of the archipelago, [d] Grand Jason, West Falklands, [e] Fox Bay, West Falklands) and are deposited in the American Museum of Natural History (AMNH) and the British Natural History Museum (BNHM).

Species	Tissue	Year	Sample size	Origin
Rockhopper penguin	Feather (adults, recent)	2009	5	New Island
	Feather (adults, historic)	1841[a]	1	BNHM
		1876[a]	2	BNHM
	Red blood cells (adults, recent)	2009	5	New Island
Gentoo penguin	Feather (adults, recent)	2007	5	New Island
	Feather (adults, historic)	1915[b]	5	AMNH
	Red blood cells (adults, recent)	2009	5	New Island
Magellanic penguin	Feather (adults, recent)	2009	5	New Island
	Feather (adults, historic)	1915 [b]	5	AMNH
	Red blood cells (adults, recent)	2009	5	New Island
Thin-billed prion	Feather (adults, recent)	2006	9	New Island
		2007	2	New Island
	Feather (adults, historic)	1915 [c]	10	AMNH
	Feather (chicks, recent)	2007	5	New Island
	Feather (adults, induced)	2007	8	New Island
	Red blood cells (chicks, recent)	2007	5	New Island
	Egg membrane	2007	6	New Island
Wilson's storm-petrel	Feather (adults, recent)	2011	4	Beauchene Island
	Feather (adults, historic)	1924[d]	2	BNHM
		1930[e]	1	BNHM

Table S2.

Reference material values measured in the analytical run carried out for this study.

S2a

	**Reference materials a** - mixtures composed of pure amino acids								
	UCD AA 1			UCD AA 2			UCD AA 3		
**AA**	**Known d^{15}N**	**Mean d^{15}N**	**SD**	**Known d^{15}N**	**Mean d^{15}N**	**SD**	**Known d^{15}N**	**Mean d^{15}N**	**SD**
Ala	-6.72	-7.09	0.48	41.13	41.40	0.27	0.83	0.36	0.68
Asp	-2.34	-2.43	0.30	-2.29	-2.26	0.93	-3.18	-3.33	0.61
Glu	-4.17	-4.18	0.77	47.57	48.08	0.41	25.86	25.60	0.85
Gly	0.82	0.99	0.50	0.73	0.97	0.57	1.62	1.54	0.64
Ile	2.53	2.76	0.32	-3.53	-3.62	0.35	-3.09	-2.76	0.61
Leu	9.24	9.71	0.39	-4.29	-5.01	0.50	4.57	3.49	0.65
Lys	-1.36	-0.71	0.34	0.47	0.80	1.09	-1.37	-0.97	1.14
Met	-1.69	-1.85	0.43	2.06	2.25	0.51	-0.47	-0.62	0.57
Phe	-1.14	-1.05	0.39	28.36	28.21	0.60	0.56	0.68	0.51
Pro	-1.44	-1.50	0.38	-0.12	0.06	0.84	26.38	26.61	0.25
Thr	-1.30	-1.40	0.82	50.19	49.44	0.48	-7.08	-7.21	1.19
Val	5.30	5.63	0.42	41.13	41.40	0.27	26.52	27.19	0.50

S2b

	**Reference materials b –** natural materials			
	RWB = baleen		MMS = fish muscle	
**AA**	**Mean d15N**	**SD**	**Mean d15N**	**SD**
Ala	14.15	0.51	27.84	0.34
Asp	11.69	0.70	29.86	0.94
Glu	16.23	0.76	29.51	0.83
Gly	5.53	0.51	4.93	0.37
Ile	16.62	0.32	29.35	0.23
Leu	14.85	0.38	27.57	0.32
Lys	5.68	1.06	8.18	1.09
Met	8.37	0.82	12.94	0.62
Phe	5.31	0.58	7.30	0.59
Pro	16.02	0.43	22.44	0.47
Thr	-15.42	1.68	-15.48	1.69
Val	17.82	0.39	29.68	0.32

QC materials

- L-Norleucine was used as an internal standard and used to calculate provisional values for each sample.
- Two mixtures composed of pure amino acids of calibrated d13C and d15N (UCD AA 1, UCD AA 2) were co-measured with samples.
- One mixture is used for final isotopic calibration of each amino acid (UCD AA 1), while the other serves as the scale normalization standard (UCD AA 2).
- Additionally, a third mixture (UCD AA 3) serves as the primary quality assurance standard (unused in corrections), while two well-described, natural materials are co-measured as secondary quality assurance materials (Here: RWB = baleen, MMS = fish muscle).

Additional Quality Assurance Criteria

- Average standard deviation of duplicate measurements will be no greater than +/- 1.25 across amino acids (within samples) and across samples (within amino acids).

- Standard deviation of individual amino acids within samples shall be no greater than +/- 1.5, except for Lys, Thr, and Tyr. Amino acids present at low concentrations in samples may not meet this criteria based on sample composition. All measures also apply to all external reference mixtures and materials.

Fig. S1. Comparison of trophic positions (TP) calculated usung the Glx and Phe approach versus the multiple amino acid approach:

TP-multi_TDF: Trophic position calculated based on Glx and Phe, as detailed in methods,

TP-7AAS: Trophic position based on all available data, namely Phe as source amino acid, and the six trophic amino acids, Glx, Ala, Leu, Ile, Pro, Asp.

TP-7AAS =1+((($\delta^{15}N_{Glx}$ + $\delta^{15}N_{Ala}$ -0.59+ $\delta^{15}N_{Leu}$ +2.93+ $\delta^{15}N_{Ile}$ -2.63+ $\delta^{15}N_{Pro}$ +1.39+ $\delta^{15}N_{Asp}$ +1.78)/6- $\delta^{15}N_{Phe}$)/5.9)

The correction values were taken from the intercepts given in Nielsen et al. (2015), ESM, Appendix C.

Nielsen JM, Popp BN, Winder M: **Meta-analysis of amino acid stable nitrogen isotope ratios for estimating trophic position in marine organisms.** *Oecologia* 2015, 178: 631-642.

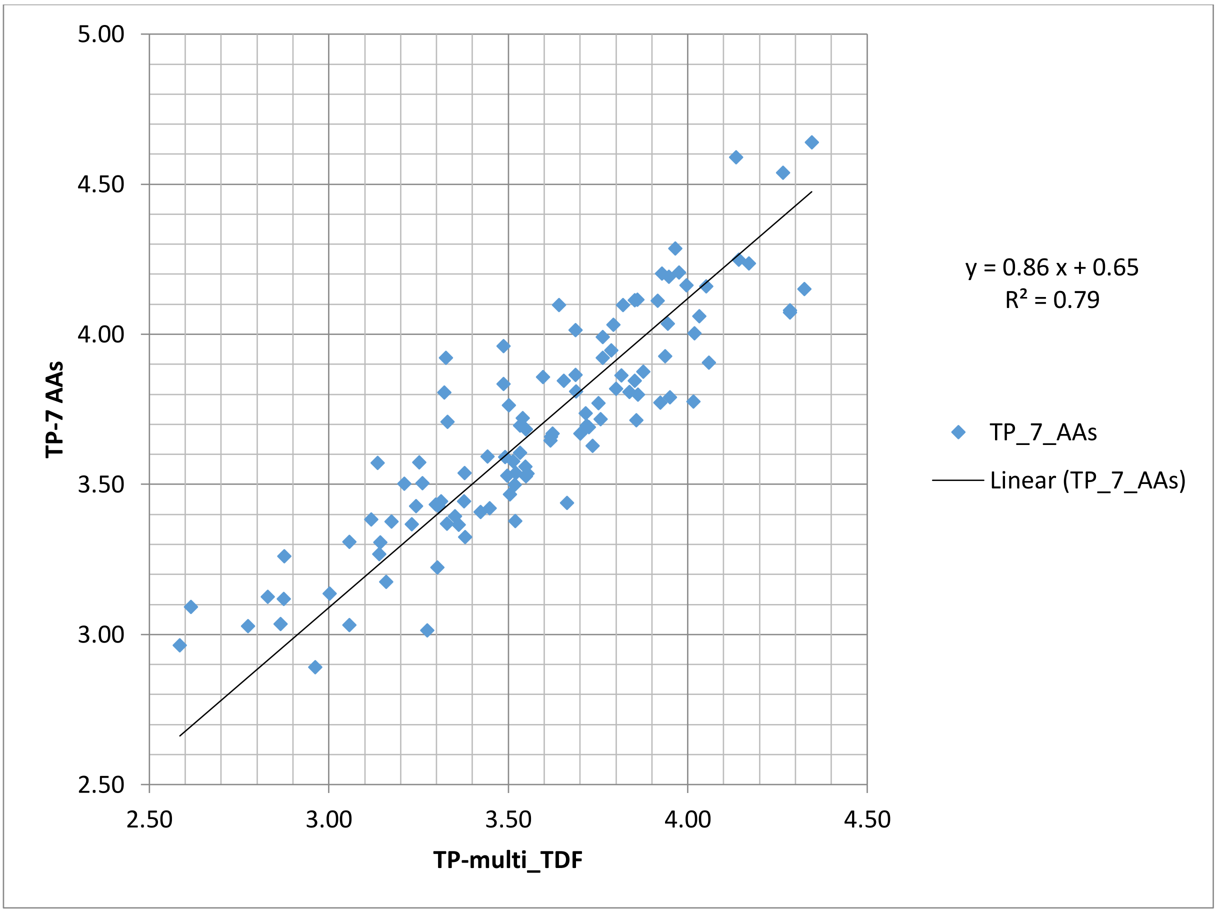

Supplement: Supplementary file 1 — Additional file 1: Table S1. Samples included in this study. All historical samples were collected in the Falkland/Malvinas area (alocality unknown, bStanley area and Beaver Island, East Falkland, coffshore waters west of the archipelago, dGrand Jason, West Falklands, eFox Bay, West Falklands) and are deposited in the American Museum of Natural History (AMNH) and the British Natural History Museum (BNHM). Table S2. Reference material values measured in the analytical run carried out for this study. Fig. S1. Comparison of trophic positions (TP) calculated usung the Glx and Phe approach versus the multiple amino acid approach. [file 12898_2020_288_MOESM1_ESM.pdf]
